# A developmental transcriptomic analysis of *Pax1* and *Pax9* in embryonic intervertebral disc development

**DOI:** 10.1242/bio.023218

**Published:** 2016-12-23

**Authors:** V. Sivakamasundari, Petra Kraus, Wenjie Sun, Xiaoming Hu, Siew Lan Lim, Shyam Prabhakar, Thomas Lufkin

**Affiliations:** 1 The Single Cell Biology Laboratory, The Jackson Laboratory for Genomic Medicine, 10 Discovery Drive, Farmington, CT 06030, USA; 2 Department of Biology, Clarkson University, 8 Clarkson Avenue, Potsdam, NY 13699, USA; 3 Computational and Systems Biology, Genome Institute of Singapore, 60 Biopolis Street, 138672, Singapore

**Keywords:** *Pax1*, *Pax9*, *Sox trio*, Intervertebral disc, TGF-B, BMP

## Abstract

*Pax1* and *Pax9* play redundant, synergistic functions in the patterning and differentiation of the sclerotomal cells that give rise to the vertebral bodies and intervertebral discs (IVD) of the axial skeleton. They are conserved in mice and humans, whereby mutation/deficiency of human *PAX1/PAX9* has been associated with kyphoscoliosis. By combining cell-type-specific transcriptome and ChIP-sequencing data, we identified the roles of *Pax1*/*Pax9* in cell proliferation, cartilage development and collagen fibrillogenesis, which are vital in early IVD morphogenesis. *Pax1* is up-regulated in the absence of *Pax9*, while *Pax9* is unaffected by the loss of *Pax1/Pax9*. We identified the targets compensated by a single- or double-copy of *Pax9*. They positively regulate many of the cartilage genes known to be regulated by S*ox5/Sox6/Sox9* and are connected to *Sox5/Sox6* by a negative feedback loop. *Pax1/Pax9* are intertwined with BMP and TGF-B pathways and we propose they initiate expression of chondrogenic genes during early IVD differentiation and subsequently become restricted to the outer annulus by the negative feedback mechanism. Our findings highlight how early IVD development is regulated spatio-temporally and have implications for understanding kyphoscoliosis.

## Abstract (precis)

**Summary:** In this study, we show the essential molecular roles of *Pax1/Pax9* in early IVD development and their connection with other chrondrogenic players – *Sox* trio, BMP and TGF B pathways.

## INTRODUCTION

The vertebral column is the fundamental infrastructure of the vertebrate body, composed of vertebral bodies (VBs) linked together by fibro-cartilaginous intervertebral discs (IVDs). During mouse embryogenesis, the vertebral column (VC) is formed from the somites through a series of precisely regulated processes. The ventro-medial cells of the somite become specified to a sclerotomal fate by Sonic hedgehog (Shh) signals derived from the notochord, which also require the maintenance of a bone morphogenetic protein (BMP)-reduced zone by BMP antagonists (Fan and Tessier-Lavigne, 1994). Shh induces the expression of *Paired-box 1* (*Pax1*), *Paired-box 9* (*Pax9*) and *Mesenchyme forkhead-1* (*Mfh1*) in the ventral somites which convey its proliferative function (Furumoto et al., 1999). The sclerotomal cells then migrate to surround the notochord and condense to form the mesenchymal prevertebrae, which is further segmented into condensed and less condensed regions along the anterior-posterior axis of the embryo, at around embryonic day (E)12.5. The former gives rise to IVD anlagen mesenchyme while the latter develop into VBs that subsequently undergo endochondral ossification. The IVD mesenchyme then differentiates into cartilaginous inner annulus (IAF) and a fibrous outer annulus (OAF) (Sivakamasundari and Lufkin, 2012; Smith et al., 2011).

*Pax1* and *Pax9* which belong to the same subfamily of *Pax* genes*,* encode transcription factors (TFs) that contain a highly conserved DNA-binding domain, the paired box (Paixao-Cortes et al., 2015). They are co-expressed in, and are critical for, the development of sclerotome-derived VBs and IVD anlagen (Neubüser et al., 1995). While both *Pax1* and *Pax9* are expressed uniformly in the condensing cells of the IVD anlagen at E12.5, with further differentiation their expression declines within the IAF and becomes restricted to the OAF by E15.5 (Wallin et al., 1994). Studies on the *Pax1 undulated* mutants and the targeted *Pax1^−/−^* and *Pax9^−/−^* knock-out (KO) mutants revealed their redundant roles in axial skeletogenesis – *Pax1* was able to fully compensate for the loss of *Pax9*, while *Pax9* was inadequate to promote normal development of the VC in the absence of *Pax1* (Wallin et al., 1994; Wilm et al., 1998). An analysis of the *Pax1/Pax9* multiple allele KO mutants uncovered their synergistic roles in axial skeleton development, demonstrating a clear gene-dosage effect of *Pax9* in the absence of *Pax1*, with increasing severity of the VC malformations, whereby *Pax1^−/−^Pax9^−/−^* mutants exhibit a complete loss of VBs and IVDs, deformed proximal parts of the ribs and a lack of caudal vertebrae. Sclerotomal cell proliferation is significantly reduced in the *Pax1*^−/−^ and *Pax1^−/−^Pax9^−/−^* embryos, hence *Pax1* and *Pax9* are believed to be essential for proliferation, but not needed for sclerotome formation. Moreover, they are postulated to have a role in early chondrogenesis in axial skeleton via regulation of processes vital for condensation of the prechondrogenic mesenchyme, such as control of cell shape/size, cell adhesion, and extracellular matrix (ECM) reorganization (Peters et al., 1999; Wallin et al., 1994).

While the functions of *Pax1/Pax9* have been hypothesized based on the phenotypic outcomes of their knock-outs, their true molecular functions, target genes and mutual regulation (or compensation) in IVD development are largely unknown. Notably, their role in axial skeletogenesis is conserved in mice and humans. Besides the high similarity of the paired-domain sequence between murine and human *Pax* genes, mutations or deficiency of *PAX1* and/or *PAX9* have been associated with Jarcho–Levine and Klippel–Feil syndromes which are characterized by kyphoscoliosis or vertebral segmentation defects that phenocopy the *Pax1^−/−^Pax9^−/−^* mouse mutants (Bannykh et al., 2003; Lopez et al., 1997). Therefore, deciphering the molecular roles of *Pax1* and *Pax9* would be valuable to understand the basis of human vertebral defects.

Here, we have identified for the first time, the *in vivo* targets of *Pax1* and *Pax9* in IVD anlagen cells to elucidate their roles during the early stages of IVD development by using enhanced green fluorescent protein (EGFP) gene targeting, fine tissue dissection and fluorescence activated cell sorting (FACS) for the specific isolation of *Pax1*- and *Pax9*-expressing cells. Using multiple allele KO embryos we identified *Pax1* and *Pax9* targets that were obscured in the single mutant embryos by the functional redundancy of *Pax1/Pax9*. Consistent with prior hypotheses, *Pax1*/*Pax9* have a role in regulating the early functions of IVD morphogenesis such as cell proliferation, adhesion, cell motion, mesenchyme condensation and ECM organization. Novel functions of *Pax1/Pax9*, namely collagen fibrillogenesis and cartilage development, were also revealed. Trends in gene expression changes of target genes with increasing loss of *Pax1/Pax9* alleles revealed potential mechanisms by which they compensate for each other. Especially, loss of *Pax9* is likely compensated for by the increased *Pax1* expression. Remarkably, 41 of the differentially expressed genes are associated with relevant axial skeletal defects. Moreover, several genes known to be regulated by the *Sox* trio (*Sox5/Sox6/Sox9*), important regulators of chondrogenesis in IVD development (Han and Lefebvre, 2008; Smits and Lefebvre, 2003), were found to be downstream targets of *Pax1* and *Pax9* as well. *In vivo* high-throughput chromatin immunoprecipitation-sequencing (ChIP-Seq) revealed critical cartilage development genes to be directly regulated by Pax9 including *Sox5* itself*.* In addition, *Pax1/Pax9* are connected with BMP and TGF-B pathways in IVD development. Finally, we also show that *Pax1/Pax9* and *Sox5* are connected by a negative feedback loop providing a potential mechanism for *Pax* gene down-regulation in the mature IAF of the IVD, and so segregating the IAF and OAF. In conclusion, we propose that *Pax1* and *Pax9* initiate robust expression of early chondrogenic genes during the earliest phase of mesenchymal differentiation in IVD development, highlighting how it is finely regulated spatio-temporally by complex transcriptional feedback loops.

## RESULTS

### *Pax1*-*EGFP* and *Pax9-EGFP* mouse lines for isolating *Pax*-specific population of cells from embryonic IVD

**Fig. 1.**
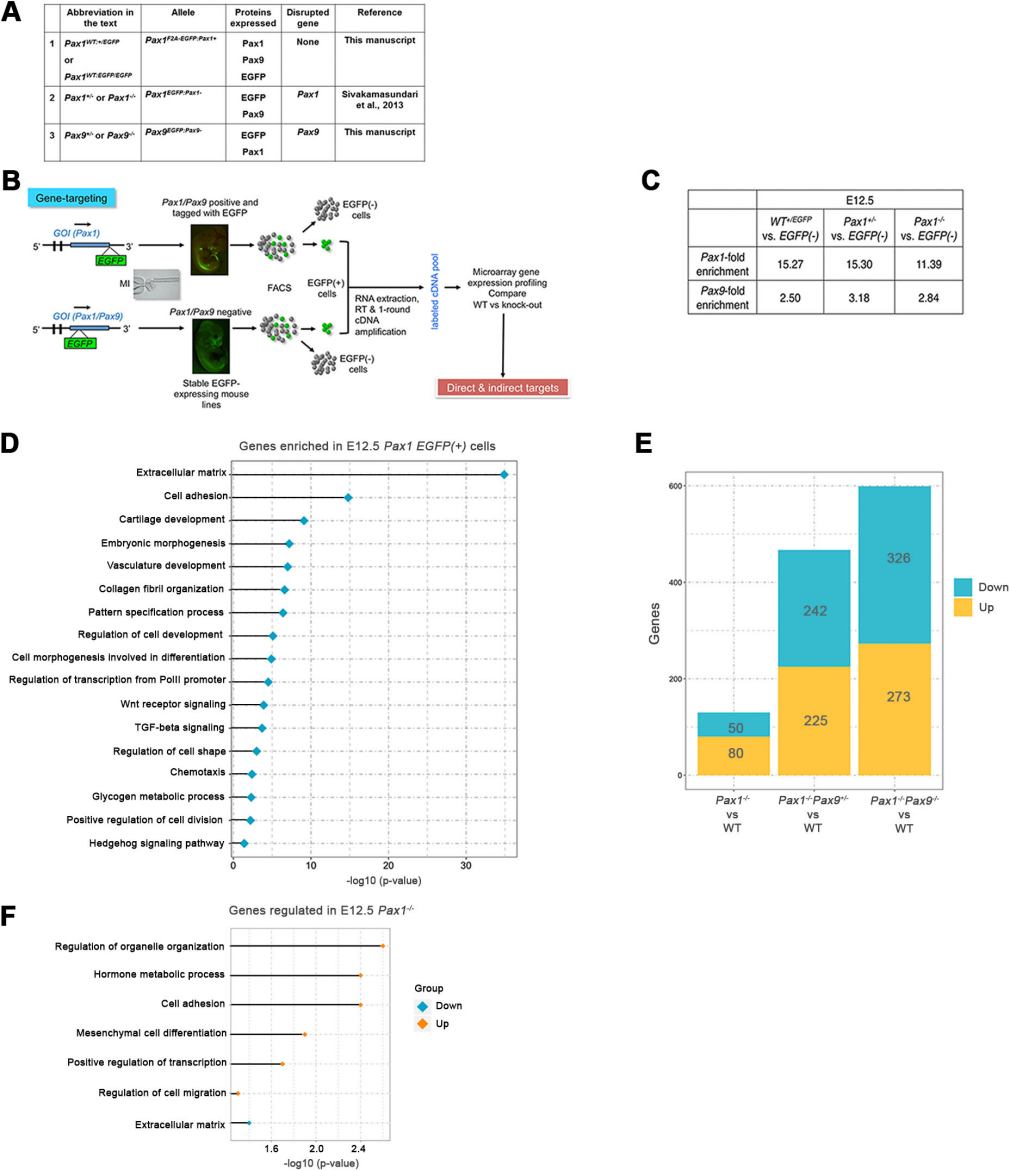
**Experimental workflow and differentially expressed genes in *Pax1/Pax9* mutants.** (A) Abbreviations of *Pax1* and *Pax9* EGFP-expressing mouse lines. (B) Diagrammatic representation of experimental workflow. (C) *Pax1* and *Pax9* fold enrichment in EGFP(+) cells compared to EGFP(−) cells from E12.5 embryos. (D) Bar chart of enriched GO terms in E12.5 *Pax1* EGFP(+) cells. (E) Number of differentially expressed genes for the various genotype comparisons. (F) Bar chart of enriched GO terms for down-regulated and up-regulated genes in E12.5 *Pax1^−/−^* embryos. GOI, gene of interest; EGFP, enhanced green fluorescent protein; FACS, fluorescence activated cell sorting; GO, gene ontology; WT, wild type.

To facilitate the isolation of *Pax1*- and *Pax9*-expressing cells from mouse embryonic tissues, we generated three knock-in transgenic mouse lines expressing *EGFP* under the control of the *Pax1* or *Pax9* regulatory elements. We used a 2A-peptide strategy to co-express EGFP downstream of a functional Pax1 protein (Fig. S1A-C). Similarly, the paired-box domain in the exon 2 of *Pax1* and *Pax9* was disrupted to generate the *Pax1* and *Pax9* knockout (KO) mouse lines (Fig. S1D-F). The generation and characterization of the *Pax1^EGFP:Pax1^*^−^ KO line (*Pax1^−/−^*) has been described already (Sivakamasundari et al., 2013). Abbreviations for mouse lines used in the text henceforth are summarized in Fig. 1A. The *Pax1^F2A−EGFP:Pax1+^* (hetero- and homozygous, henceforth referred to as *Pax1^WT:+/EGFP^* and *Pax1^WT:EGFP/EGFP^*, respectively) and *Pax9^+/EGFP:Pax9−^* (called henceforth *Pax9^+/−^*) mice were viable and fertile while the *Pax9^EGFP:Pax9−/EGFP:Pax9−^* (called henceforth *Pax9^−/−^*) exhibited post-natal lethality as previously reported. These mouse lines expressed *EGFP* in the correct *Pax1*- or *Pax9*-specific domains (Fig. S2). Immunohistochemistry (IHC) analyses confirmed the absence of Pax9 protein in the *Pax9^−/−^* embryos (Fig. S3C).

In order to investigate the functions of *Pax* genes and their dosage compensation during IVD development, *EGFP*-positive cells were isolated from dissected vertebral column (VC) tissue at E12.5 via FACS and subsequent gene expression analyses were performed (Fig. 1B). In the population of VC cells isolated from E12.5 *Pax1^WT:+/EGFP^*, *Pax1^+/−^* and *Pax1^−/−^* embryos, *Pax1* wild-type or mutant transcripts were highly enriched in the EGFP(+) vs EGFP(−) cells, indicating a successful enrichment for the correct cell population (*Pax1* expressing) from these embryos (Fig. 1C and Table S1). *Pax9* was also enriched in these EGFP(+) cells albeit not to the same extent as *Pax1* (Fig. 1C). Gene expression profiling of EGFP(−) versus EGFP(+) cells from *Pax1^WT:+/EGFP^* revealed 744 down-regulated and 1052 up-regulated genes in the EGFP(+) cells relative to the EGFP(−) cells [Table S1; fold change (FC)≥1.5 and *P*<0.05]. Gene ontology (GO) enrichment analysis revealed ‘biological processes’ categories such as extracellular matrix (ECM), cell adhesion, cartilage development, collagen fibril organization, pattern specification, chemotaxis, cell shape and cell division to be over-represented. Genes involved in Wnt, TGF-B and hedgehog signaling pathways were also enriched (Fig. 1D) (DAVID; Huang et al., 2009b). Several other genes known to be expressed in the IVD anlagen or involved in the sclerotome differentiation and/or IVD development, such as *Meox1*, *Sox5*, *Foxc2*, *Adamtsl2*, *Fmod* and *Tgfbr2*, were enriched in EGFP(+) cells (Table S1) (Furumoto et al., 1999; Mankoo et al., 2003; Smits and Lefebvre, 2003; Sohn et al., 2010).

### *Pax1* and *Pax9* regulate proliferation, mesenchyme condensation and IVD development genes

At E12.5 the only morphological defects apparent in the *Pax1^−/−^* embryos were a loss of VB and IVD cells mainly in the lumbo-sacral region owing to partially redundant functions of the paralogous gene *Pax9* (Sivakamasundari et al., 2013; Wilm et al., 1998). Analysis of *Pax1*+ cells from the E12.5 *Pax1^−/−^* vs *Pax1^WT:+/EGFP^* IVD anlagen, revealed 50 down- and 80 up-regulated genes in *Pax1^−/−^* (FC≥1.5 and *P*<0.05) (Fig. 1E; Table S2). Down-regulated genes were enriched for ECM, while genes involved in organelle organization, cell adhesion, mesenchymal cell differentiation, transcriptional regulation and cell migration were up-regulated (Fig. 1F).

**Fig. 2.**
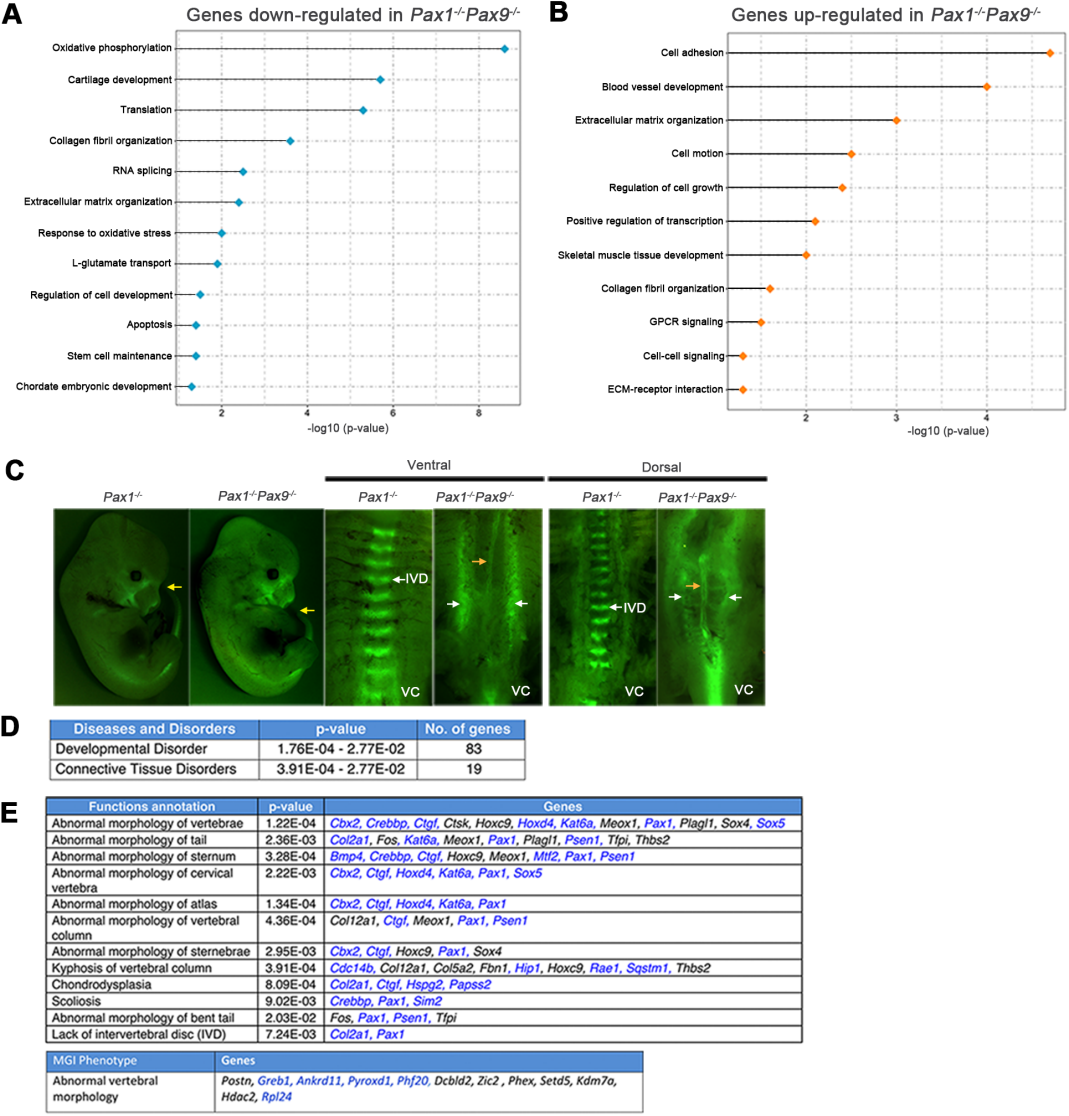
**Gene ontology and Ingenuity Pathway Analyses (IPA) of differentially expressed genes in *Pax1/Pax9* mutants.** (A,B) Bar chart of enriched GO terms for down-regulated (A) and up-regulated genes (B) in E12.5 *Pax1^−/−^Pax9^−/−^* embryos. (C) *Pax1^−/−^Pax9^−/−^* embryos exhibited shortened tail phenotype compared to *Pax1^−/−^* embryos. Yellow arrows indicate tail tip. Fluorescence was seen in the IVD of E14.5 *Pax1^−/−^Pax9^+/+^*embryos in a regular metameric fashion. In the *Pax1^−/−^Pax9^−/−^* embryos, the fluorescing cells were mis-localized to the sides of the embryo with the notochord exposed in the middle (orange arrow). White arrows indicate *Pax1/Pax9* lineage cells expressing EGFP in the IVD, detected by the presence of GFP expression. (D) Number of differentially expressed genes in E12.5 *Pax1^−/−^Pax9^−/−^* associated with the respective disease and disorder terms identified via IPA. (E) Genes differentially expressed in E12.5 *Pax1^−/−^Pax9^−/−^* that are associated with the respective axial skeletal defects, identified via IPA (top) and MGI phenotype (bottom). Genes down-regulated in *Pax1^−/−^Pax9^−/−^* respective to WT are in blue. GO, gene ontology; VC, vertebral column; IVD, intervertebral disc anlagen.

We subsequently analyzed the *Pax1^−/−^Pax9^+/−^* and *Pax1^−/−^Pax9^−/−^* compound mutants to uncover the obscured targets of *Pax1* and *Pax9*. There were 599 genes differentially expressed (326 down; 273 up) in *Pax1^−/−^Pax9^−/−^* (versus *Pax1^WT:+/EGFP^*) embryos. This is a 4.6-fold increase in the number of affected genes compared to *Pax1^−/−^*. In *Pax1^−/−^Pax9^+/−^* versus *Pax1^WT:+/EGFP^*, 467 genes were differentially expressed, consistent with the less severe VC phenotype of *Pax1^−/−^Pax9^+/−^* mutants (Fig. 1E; Fig. S2, Table S3) (Peters et al., 1999). GO terms enriched in the down-regulated targets of *Pax1^−/−^Pax9^−/−^* include oxidative phosphorylation, cartilage development, apoptosis and stem cell maintenance. Notable GO terms over-represented in up-regulated targets were cell adhesion, blood vessel development, cell motion, skeletal muscle tissue development, GPCR signaling and cell-cell signaling. Common categories significantly enriched in both up- and down-regulated targets were ECM organization and collagen fibril organization (Fig. 2A,B). Ingenuity Pathway Analysis (IPA; Ingenuity® Systems, www.ingenuity.com) also predicted ‘proliferation of cells’ to be decreased (z score=−2.524) and 85 proliferation-associated genes were differentially expressed in *Pax1^−/−^Pax9^−/−^* versus *Pax1^WT:+/EGFP^*, consistent with a significant shortening of the tail in the E12.5 *Pax1^−/−^Pax9^−/−^* embryos, which worsened during later developmental stages (Fig. 2C; Table S4, Fig. S2O-Q). This corroborated prior findings of a significant decrease in proliferation in the tail region of E12.5 *Pax1^−/−^Pax9^−/−^* embryos (Peters et al., 1999). In addition, for the first time, we describe a mis-localization of the normally ventro-medially located *Pax1/Pax9*-specific cells to the lateral regions of the VC at E14.5. No complete VBs or IVD structures were seen at this stage (Fig. 2C).

‘Developmental Disorder, Cell Morphology, Cellular Assembly and Organization’ was the top associated network (score=40) in IPA, with 83 genes associated with ‘Developmental Disorder’ (*P*=1.76E–04-2.77E–02) and 19 genes associated with ‘Connective Tissue Disorders’ (*P*=3.91E–04-2.77E–02) (Fig. 2D; Table S4); 41 of the differentially expressed genes were associated with relevant skeletal defects seen in the *Pax1^−/−^Pax9^−/−^* mutants, such as kyphosis, scoliosis, lack of IVD, abnormal morphology of VC/tail/cervical vertebrae/sternum and chondrodysplasia (Fig. 2E; Tables S4 and S5). These results suggest that *Pax1* and *Pax9* regulate genes involved in processes essential for proper mesenchymal condensation – cell adhesion, ECM organization and cell migration during IVD development (Hall and Miyake, 2000).

### Gene dosage effect of *Pax1* and *Pax9* at molecular level

**Fig. 3.**
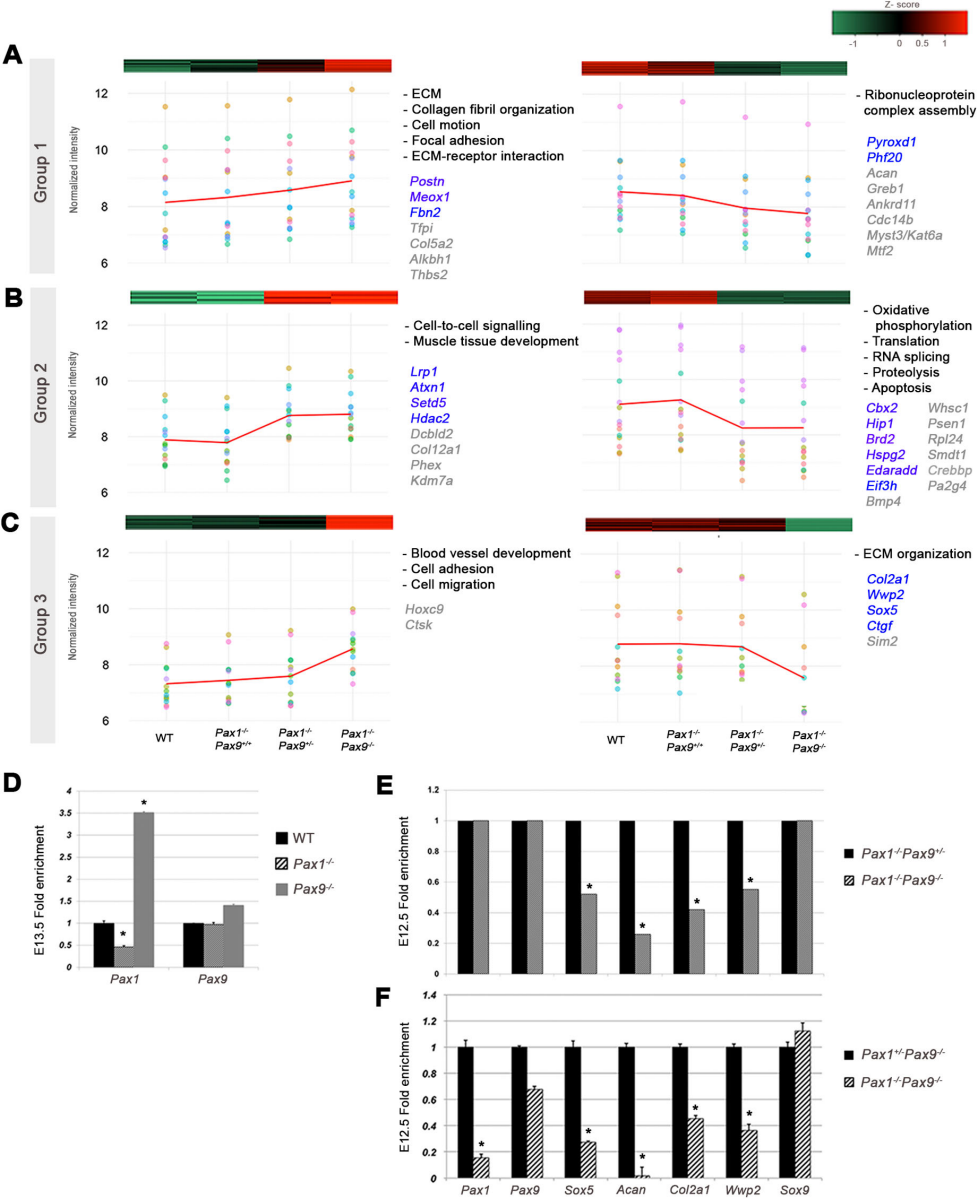
**Gene dosage effect of *Pax1*/*Pax9* on down-stream targets and regulation of cartilage development genes.** (A-C) Differentially expressed genes that show: (A) Group1, gradual decrease or increase in expression with increasing loss of *Pax1/Pax9* alleles; (B) Group 2, genes that require two copies of *Pax9* to maintain their normal expression levels; and (C) Group 3, genes showing a significant change upon the loss of the last copy of *Pax9*. Mean trend of all genes in each subgroup is depicted as red line for respective groups; individual gene trends are shown as different colored dots. Enriched GO terms for each category indicated on the right of respective graphs. Genes with abnormal skeletal phenotype are shown in gray, of which direct Pax9 targets are in blue for the respective groups. (D) qPCR fold change of *Pax1* and *Pax9* in E13.5 WT, *Pax1^−/−^* and *Pax9^−/−^*. (E) Fold enrichment of selected cartilage development genes from microarray analysis of E12.5 *Pax1^−/−^Pax9^−/−^* versus *Pax1^−/−^Pax9^+/−^*. (F) qPCR fold-enrichment of cartilage development genes in E12.5 *Pax1^−/−^Pax9^−/−^* vs. *Pax1^+/−^Pax9^−/−^*. qPCR, quantitative PCR; FC, fold change; * significant FC ≥1.5, Student's *t*-test; error bars indicate s.e.m.

To understand the gene dosage effect of *Pax1* and *Pax9* at a molecular level, we analyzed the trends in expression changes of their targets upon gradual loss of *Pax1* and *Pax9* alleles. Majority of the genes could be categorized into three main groups (Fig. 3A-C).

Group 1 (118 down, 108 up) genes showed a gradual decrease or increase in expression with increasing loss of *Pax1/Pax9* alleles, whereby a significant change in expression level (FC>1.5) occurred only upon the loss of all four alleles. This was consistent with the observed increasing severity of the mouse phenotype with increasing loss of *Pax1* and *Pax9* alleles. Genes found in this group were mostly ECM-related and included *Acan*. Notably, chromatin remodeling and acetylation factors like *Ep300* and *Phf20* were also found in this category (Fig. 3A; Tables S5 and S6).

Group 2 (166 down, 109 up) consisted of genes which showed a significant change only upon the loss of three alleles (*Pax1^−/−^Pax9^+/−^*), but no further significant change upon the loss of the last copy of *Pax9* (*Pax1^−/−^Pax9^−/−^*). This suggests that two copies of *Pax9* were essential to maintain the normal expression level of these genes in the absence of *Pax1.* This group encompassed oxidative phosphorylation genes among other ECM genes like *Hspg2* and *Cspg2* and cartilage development gene *Bmp4* (Fig. 3B; Tables S5 and S6).

Group 3 (28 down, 31 up) genes showed a significant change only upon the loss of the last copy of *Pax9* in the absence of *Pax1*. Intriguingly, this included several key cartilage development and collagen fibrillogenesis genes such as *Col2a1*, *Sox5*, *Wwp2*, *Sim2, Ctgf* and *Dpt* (Fig. 3C; Tables S5 and S6). These genes could be too crucial to be compromised, hence maintained at appropriate levels even by the last copy of *Pax9*.

### Regulation of *Pax1* and *Pax9*

We next examined the expression levels of *Pax1* and *Pax9* in the various mutants to understand their compensation mechanism. *Pax1* was down-regulated in *Pax1^−/−^* mutant, but *Pax9* remained unchanged in *Pax9^−/−^* (Fig. 3D; Table S4). This indicated that Pax1 could auto-regulate itself, unlike Pax9. Indeed, *Pax1* was downregulated in the comparison between *Pax1^+/−^Pax9^−/−^* and *Pax1^−/−^Pax9^−/−^*, revealing that a single copy of *Pax1* could regulate itself (Fig. 3F); however, *Pax9* was not considerably altered in any of the compound mutants (Fig. 3E,F; Table S3). This corroborates prior observations that *Pax9* expression is not dependent on *Pax1* or *Pax9* (Neubüser et al., 1995).

*Pax1* did not significantly change between *Pax1^−/−^Pax9^+/−^* and *Pax1^−/−^Pax9^−/−^* (Fig. 3E; Table S3). Moreover, there was only a slight decrease (1.5-fold) in *Pax1* between *Pax1^−/−^Pax9^+/+^* and *Pax1^−/−^Pax9^−/−^* (Table S3). This suggested that at least two copies of *Pax9* was required to even have a minute effect on *Pax1*. We also did not observe any significant change in *Pax9* level or spatial expansion of its expression domain in *Pax1*^−/−^ mutants based on microarray (Table S2) and IHC analyses (Fig. S3).Yet, *Pax1* expression was significantly up-regulated in E13.5 *Pax9^−/−^* mutants (Fig. 3D).

Together these data suggest that *Pax9* is unable to compensate for the loss of *Pax1* by up-regulating itself at E12.5 and E13.5, while *Pax1* may compensate for the loss of *Pax9* by altering its own expression level via a positive auto-feedback mechanism.

### *Pax1* and *Pax9* redundantly regulate key cartilage development genes

Closer inspection of genes regulated by *Pax1* and *Pax9* revealed key cartilage development genes like *Sox5*, *Acan*, *Col2a1* and *Wwp2* to be within the top 30 positively regulated targets of *Pax1* and *Pax9*. KO mutants of these four genes are known to exhibit axial skeletal and craniofacial defects similar to *Pax1^−/−^Pax9^−/−^* mutants (Aszódi et al., 1998; Smits and Lefebvre, 2003; Watanabe et al., 1997; Zou et al., 2011).

Removal of the last copy of *Pax9* in the absence of *Pax1* (*Pax1^−/−^Pax9^−/−^* vs *Pax1^−/−^Pax9^+/−)^* revealed its positive regulatory effect on *Sox5*, *Acan*, *Col2a1* and *Wwp2* (Fig. 3E; Table S3). Quantitative PCR (qPCR) of FACS-sorted cells of *Pax1^+/−^Pax9^−/−^* and *Pax1^−/−^Pax9^−/−^* showed a similar trend, whereby all four targets were positively regulated by a single copy of *Pax1* in the absence of *Pax9*, affirming that both *Pax1* and *Pax9* were independently capable of up-regulating these targets (Fig. 3F). We validated some of these targets by sectioned *in situ* hybridization (Fig. S5.2).

While these targets are also known to be positively regulated by *Sox9* (Akiyama et al., 2002; Bell et al., 1997; Han and Lefebvre, 2008; Nakamura et al., 2011), a key regulator of chondrogenesis, we did not observe significant changes in *Sox9* expression levels in *Pax1^−/−^Pax9^−/−^* versus *Pax1^WT:+/EGFP^* (Table S3) and *Pax1^−/−^Pax9^−/−^* versus *Pax1^+/−^Pax9^−/−^* comparisons (Fig. 3F). This suggests that the observed *in vivo* changes in expression levels of these genes are *Pax1-* and *Pax9*-dependent and not caused by changes in *Sox9* levels, indicating the existence of a potential parallel *Sox9* independent mechanism.

### *Pax9* directly regulates chondrogenic genes essential for IVD morphogenesis

These observations prompted us to inspect if *Pax9* was capable of directly regulating these critical cartilage development and collagen fibrillogenesis genes to compensate for the loss of the *Pax1*.

**Fig. 4.**
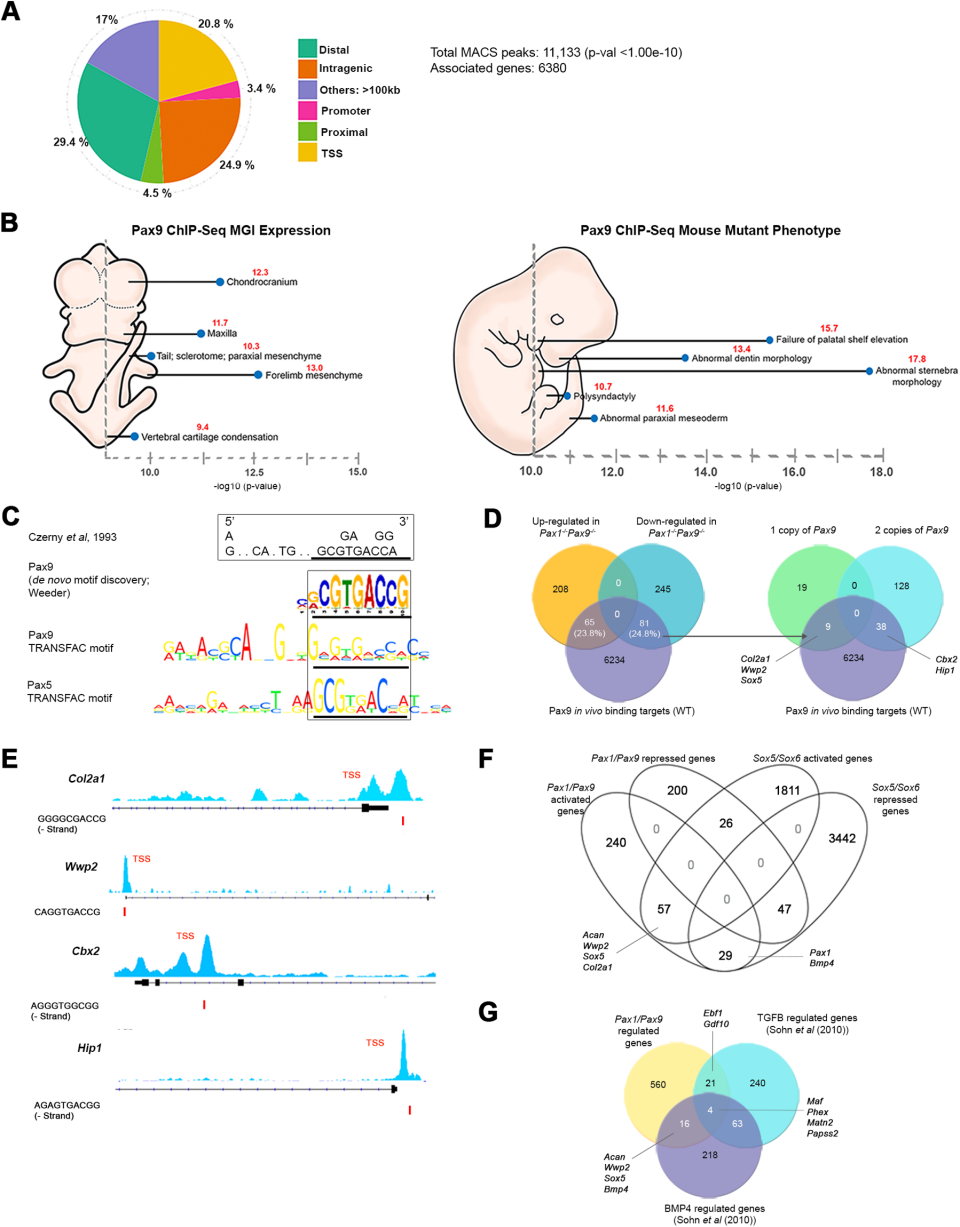
**Pax9 direct *in vivo* targets and genes co-regulated by *Pax*, *Sox*, Bmp4 and TGF-B.** (A) Pax9 binding site distribution in E12.5 WT vertebral column cells. (B) Gene ontology analyses of genes associated with Pax9 binding sites: MGI expression profile and mouse mutant phenotype. (C) *De novo* Pax9 motif discovered in *in vivo* Pax9 ChIP-Seq, that resembles the reported 3′ half site of Pax family of transcription factors. (D) Overlap of Pax9 binding associated targets with differentially expressed genes in the *Pax1^−/−^Pax9^−/−^* mutant. Overlap of genes regulated by single or two copies of Pax9 with Pax9 binding associated genes shown on right. (E) Pax9 binding peaks, visualized using IGV, at the TSS of *Col2a1*, *Wwp2*, *Cbx2* and *Hip1*. Pax9 motif at the TSS identified via FIMO analysis is shown below as a red bar. (F) Overlap of genes regulated by *Pax1/Pax9* and *Sox5/Sox6* in the IVD anlagen. (G) Overlap of genes regulated by *Pax1/Pax9*, TGF-B pathway and *Bmp4*. TSS, transcriptional start site; IGV, Integrative Genomics Viewer.

Therefore, we assessed the *in vivo* binding profile of *Pax9* by chromatin immunoprecipitation sequencing (ChIP-Seq) on dissected E12.5 WT VC tissues. There were 11,133 Pax9 binding loci which were associated with 6380 genes (Table S7). Majority of the Pax9 binding sites were found in the distal (29.4%), followed by intragenic (24.9%), TSS (20.8%), >100 kb from TSS region (17.0%), proximal (4.5%) and promoter regions (3.4%) (Fig. 4A). GO analysis revealed that the genes associated with Pax9 binding sites were enriched for *Pax9* expression domains: maxilla, vertebral cartilage, chondrocranium, paraxial and forelimb mesenchyme; and the relevant mouse mutant phenotypes: abnormal dentin/pterygoid process, abnormal palate shelf elevation, abnormal sternebra morphology and paraxial mesoderm and polysyndactyly (Fig. 4B) (McLean et al., 2010). *De novo* motif discovery identified a Pax9 motif, 5′ CGCGTGACCG 3′, that resembled the previously reported 3′ half site for the Pax family of TFs (Fig. 4C) (Czerny et al., 1993). Analysis using Centrimo also revealed Pax DNA binding domains (DBD) to be centrally enriched (Fig. S4A,B) (Bailey and Machanick, 2012). It is noteworthy that the Pax DBDs are highly similar, so various Pax TFs appear to be enriched.

In addition, 24.4% (146 genes) of the differentially expressed genes in *Pax1^−/−^Pax9^−/−^* had Pax9 binding sites associated with them. This corresponds to 24.8% of the down-regulated and 23.8% of the up-regulated targets (Fig. 4D). Of the down-regulated genes, nine of them were directly regulated by a single copy of Pax9 while 38 were regulated by two copies of Pax9 (Fig. 4D). This included *Col2a1*, *Wwp2*, *Cbx2*, *Hip1*, *Sox5* but not *Acan*, indicating that the latter is an indirect target of *Pax9*. These binding regions also possessed the Pax9 *de novo* motif (*P*<0.005) (Fig. 4E). Since Pax9 is capable of directly regulating these genes it explains why their expression levels do not significantly change in the absence of only *Pax1*, and reveals how it compensates for these genes (Fig. 3A-C,E).

### *Sox5* and *Sox6* negatively regulate *Pax1* in IVD anlagen cells

**Fig. 5.**
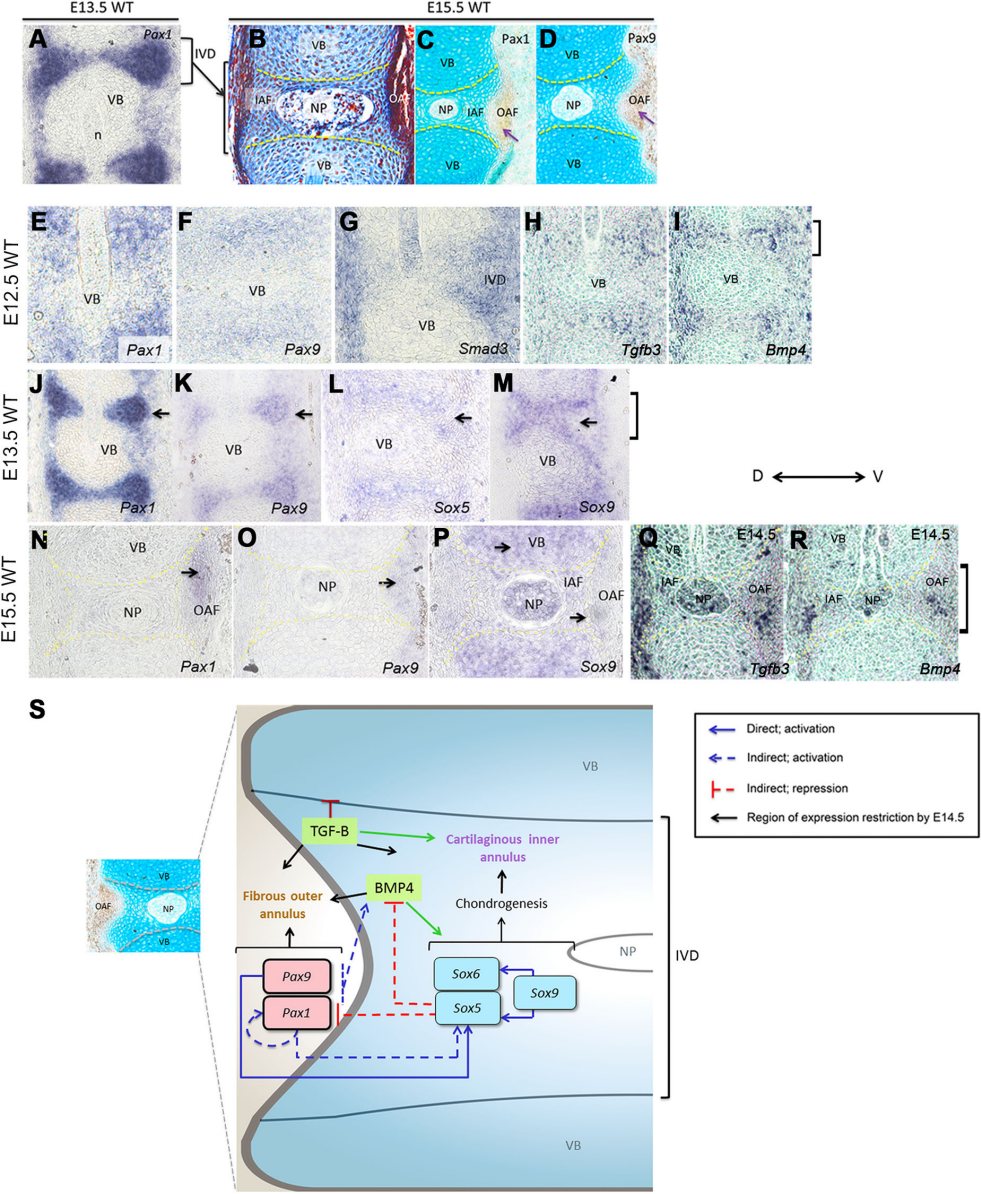
**IVD expression of *Pax* and *Sox* genes and proposed model.** (A) Sagittal section of E13.5 WT vertebral column showing *Pax1* expression in the IVD anlagen detected by SISH assay. (B) Mallory's tetrachrome staining of E15.5 WT vertebral column; IAF is stained blue and OAF is stained dark red. (C,D) Immunohistochemistry and Alcian blue staining of vertebral column showing (C) Pax1 and (D) Pax9 protein expression restricted to the OAF at E15.5. (E-I) Expression of *Pax1*, *Pax9*, *Smad3*, *Tgfb3* and *Bmp4* in the IVD anlagen at E12.5. (J-M) Expression of *Pax1*, *Pax9*, *Sox5* and *Sox9* in the IVD anlagen at E13.5. (N-R) Expression of *Pax1*, *Pax9*, *Sox9* at E15.5 and *Tgfb3* and *Bmp4* at E14.5 in the IVD. (S) Proposed negative feedback loop mechanism between *Pax*, *Sox*, Bmp4 and TGF-B pathway at E12.5. Black arrows indicate the final site of expression at E14.5. Brackets indicate IVD region. VB, vertebral body; n, notochord; IVD, intervertebral disc; NP, nucleus pulposus; IAF, inner annulus fibrosus; OAF, outer annulus fibrosus; WT, wild type; SISH, sectioned *in situ* hybridization; D, dorsal; V, ventral.

*Sox5* and *Sox6* are important in IVD morphogenesis, whereby they play redundant but important roles in up-regulating major cartilage ECM genes like *Acan* and *Col2a1* for the timely maturation of chondroblasts and promoting inner annulus differentiation (Smits and Lefebvre, 2003). Also, *Sox5* is co-expressed with *Pax1/Pax9* in the VC at E12.5 and E13.5 (Fig. 5; Table S1). Hence, we investigated previously generated *Sox5^−/−^* and *Sox6^−/−^ EGFP^+^ WT* and *KO* mutants (data not shown) made in an identical manner to the *Pax1* and *Pax9* alleles described here and found *Pax1* up-regulated (1.60-fold) in the E13.5 IVD cells of *Sox5^−/−^Sox6^−/−^* embryos but not in the individual *Sox5^−/−^* or *Sox6^−/−^* mutants (GSE33173; Fig. 4F; Table S8). This suggests a synergistic repressive effect of both *Sox* genes on *Pax1*, while *Pax9* expression was unchanged in the *Sox5^−/−^* or *Sox6^−/−^* or *Sox5^−/−^Sox6^−/−^* mutants.

Considering that *Pax1* and *Pax9* have an activating effect on *Sox5*, and *Sox5*/*Sox6* synergistically repress *Pax1*, and positive regulation of *Sox5/Sox6* by *Sox9* is well-established (Akiyama et al., 2002), we hypothesize that these *Pax* and *Sox* genes are connected in a negative feedback loop during VC development (Fig. 5S). We posit that *Pax1*/*Pax9* are downregulated in the cartilaginous IAF by this negative feedback mechanism, possibly playing a role in the segregation of IAF from OAF by E15.5.

### *Pax1/Pax9* regulation of IVD development is intertwined with BMP and TGF-B pathways

We found both *Pax1/Pax9* and *Sox5/Sox6* regulated *Bmp4* indicating the involvement of BMP pathway in IVD development (Fig. S5.2A). A study by Sohn et al. (2010) showed that BMP signaling activated the *Sox* trio and cartilage genes (*Acan*,* Wwp2*) in the sclerotome, while TGF-B signaling regulated IVD markers (*Fmod* and *Adamtsl2*) and maintained the boundary between vertebrae and IVD in the axial skeleton (Sohn et al., 2010). Also, studies on *Tgbr2* conditional KO mutants have shown that TGF-B signaling is required to define the boundary between VB and IVD and to promote differentiation of annulus fibrosus from the sclerotome (Baffi et al., 2006, 2004; Sohn et al., 2010). Therefore we probed in our data if *Pax1/Pax9* regulate Bmp4 or TGF-B regulated genes.

Using data from Sohn et al. (2010) performed at E12.5, we compared our list of *Pax1/Pax9* regulated genes with the list of genes regulated by BMP and TGF-B pathway. We found some of the BMP and TGF-B pathway genes were also regulated by *Pax1/Pax9* (Fig. 4G; Table S9). Importantly, *Sox5*, *Acan* and *Wwp2* were activated by both *Pax1/Pax9* and BMP pathway, while *Papss2* was activated by BMP, TGF-B pathways and *Pax1/Pax9* (Fig. 4G; Table S9). Also, VB enriched genes identified by Sohn et al. (2010) were repressed by both *Pax1/Pax9* and TGF-B (*Ebf1*, *Gdf10*, *Alcam*, *Nr2f2*). Indeed, from our Pax9 ChIP-seq data, we found Smad1 and Smad2 motifs to be centrally enriched (within 100 bp) in the Pax9 bound regions (Fig. S4C) (Bailey et al., 2009; Bailey and Machanick, 2012). Also, *Smad3*, *Tgfb3* and *Bmp4* were all expressed in the IVD anlagen at E12.5 (Fig. 5). This affirmed that *Pax1/Pax9*, BMP and TGF-B pathways were interlinked in the regulation of some of the IVD genes.

The distinction between IAF and OAF happens only at E14.5 onwards, and is more apparent at E15.5 (Fig. 5A-D). To assess whether *Pax*, BMP and TGF-B pathways played a role in the distinction between IAF and OAF, we performed some sectioned *in situ* hybridization (SISH) and also mined the database of gene expression at E14.5 (EURexpress database, www.eurexpress.org; Diez-Roux et al., 2011). Among the 41 genes regulated by *Pax1/Pax9*, TGF-B and *Bmp4*, a majority of those activated by *Pax1/Pax9* at E12.5 were expressed in the IAF later at E14.5, while those repressed were mainly expressed in OAF and VB at E14.5 (Table S9, EURexpress database). On the contrary, several of the targets activated at E12.5 by *Pax1/Pax9* alone were expressed highly in the E14.5 OAF as well (Fig. S5.1, Fig. S5.2). These indicated that *Pax1/Pax9* on their own were capable of activating both IAF and OAF genes, while in combination with BMP and TGF-B pathways, and were mainly involved in promoting IAF.

Notably, *Pax1/Pax9*, *Bmp4* and *Tgfb3* were restricted to OAF, while *Bmpr2*, *Tgfbr1*, *Smad2* and *Smad3* were all expressed in both IAF and OAF at E14.5-E15.5, suggesting that *Pax1/Pax9*, BMP and TGF-B signaling were active in OAF at E14.5 and may play a role in further OAF differentiation (Fig. 5; EURexpress database; Table S9).

## DISCUSSION

The importance of *Pax1* and *Pax9* in axial skeletogenesis was demonstrated more than a decade ago using KO mouse models, but not as much progress has been made since to decipher their molecular roles (Hall and Miyake, 2000). Our genome-wide study is the first to identify the *in vivo* target genes of *Pax1* and *Pax9* in the early embryonic IVD in a cell-type-specific manner. The E12.5 developmental stage was chosen as this is when the IVD anlagen is first formed. We have identified genes coexpressed in *Pax1* expressing cells to be involved in the *Wnt*, *Tgf-B* and *hedgehog* signaling pathways. These pathways are critical in a vast number of cellular processes relevant to the formation of mesenchymal condensations and chondrogenesis/osteogenesis in early IVD development (Buttitta et al., 2003; Ling et al., 2009; Sohn et al., 2010). Furthermore, our analysis of *Pax1^−/−^* and *Pax1/Pax9* compound mutants revealed the roles of *Pax* genes in processes essential for mesenchymal condensation – a pre-requisite for the formation of skeletal elements via endochondral ossification well-known to be disrupted in *Pax* mutants (Wallin et al., 1994).

The small number of differentially expressed genes in the E12.5 *Pax1^−/−^* embryos indicated that the complete repertoire of targets were being masked because of compensation by *Pax9*, consistent with the mild phenotype whereby a shortening of tail was obvious only from E13.5 onwards (Sivakamasundari et al., 2013). Analysis of the compound mutants embryos revealed several more targets of *Pax1* and *Pax9*, fitting the observed severity of the phenotype with increasing loss of *Pax1* and *Pax9* alleles. One of the striking defects reported for *Pax1^−/−^Pax9^−/−^* mutants were reduced cell proliferation in the lumbo-sacral region (Peters et al., 1999). In our study, a significant number of cell proliferation genes were affected only upon the KO of 3/4 or 4/4 (all) alleles but not in the *Pax1^−/−^* (2/4 alleles). This correlates with the shortened tail, evident as early as E12.5, and the loss of the medially located IVD and VB cells in the double-null mutants. These defects already prefigure the future adult *Pax1^−/−^Pax9^−/−^* mutants, which exhibit a complete lack of sacral elements, IVD or VB structures (Peters et al., 1999). Alterations to the cell-adhesion and cell motion genes may also play a role in the observed mis-localization of *Pax1/Pax9* cells to the lateral regions of the VC in the *Pax1^−/−^Pax9^−/−^* embryos. Besides proliferation, oxidative phosphorylation genes were affected in the double-null mutants. It has been shown that disruption of oxidative phosphorylation affects cell viability and proliferation in neural progenitors (Lee et al., 2013). It is likely that this could have partly contributed to the overall decrease in proliferation and/or cell viability of *Pax1/Pax9* deficient cells. Highly proliferating cells such as the cells of the somites are believed to be particularly sensitive to changes in rates of protein synthesis (Oliver et al., 2004). Down-regulation of several ribosomal genes may have an effect on translation in general, which could also have contributed to the reduced cell proliferation. Furthermore, we found several of the *Pax1*/*Pax9* regulated genes to be associated with axial skeleton defects in humans that phenocopy the *Pax1^−/−^Pax9^−/−^* mouse mutants. For instance, the human ortholog of *Acan*, *Col2a1*, and *Col11a1* are linked to spondyloepiphyseal dysplasia, osteoarthritis, disc degeneration (Nakane et al., 2011; Sahlman et al., 2001), and susceptibility to lumbar disc herniation (Mio et al., 2007).

Beyond understanding the molecular roles of *Pax1* and *Pax9*, we aimed to address how they compensate for each other. We have uncovered the gene dosage effect of *Pax1* and *Pax9* at a molecular level by identifying the groups of genes requiring all four copies of *Pax1/Pax9*, two copies of *Pax9* or only one copy of *Pax1* or *Pax9*. We delved deeper to understand if expression of *Pax1* and *Pax9* themselves changed to cater to the dosage compensation. To maintain the required dosage of Pax proteins for gene regulation, it is expected that *Pax1* or *Pax9* should be up-regulated in the absence of the other protein. We observed that *Pax1* is up-regulated in the absence of Pax9, suggesting that it may compensate for the required dosage of Pax by up-regulating its expression via auto-regulation. Only upon the loss of one more copy of *Pax1* (*Pax1^+/−^Pax9^−/−^*), its dosage likely becomes critical. With only one copy of *Pax1*, the system may be sufficiently deprived by diminishing levels of *Pax1* being produced, thus resulting in the onset of more severe vertebral defects in *Pax1^+/−^Pax9^−/−^*; however, *Pax9* was not up-regulated in the absence of Pax1 protein which could explain its inability to completely rescue VC defects in *Pax1^−/−^*. These observations support the existing hypothesis that *Pax1* is the more dominant player since it fully rescues the vertebral phenotype in the *Pax9^−/−^* mutant, unlike *Pax9* (Peters et al., 1999). We postulate that in the absence of *Pax1*, Pax9 maintains the levels of vital IVD morphogenesis genes (*Sox5*, *Col2a1*, *Wwp2*) owing to its ability to directly regulate them. Also, we speculate that Pax1 and Pax9 may be capable of binding to each other's sequences, as their paired domains are highly similar with only three amino acid changes (Neubüser et al., 1995). In such a scenario, competition would exist between Pax1 and Pax9 under wild-type conditions, whereby the amount of protein, partner proteins (cofactors), chromatin accessibility and/or the time at which the proteins are expressed may determine whether Pax1 or Pax9 bind the specific site and their affinity in binding. Temporal differences in the emergence of *Pax1* and *Pax9* expression exist, whereby *Pax1* is expressed in the de-epithelializing somites at a slightly earlier time-point (E8.5) than *Pax9* (E9.0) (Neubüser et al., 1995; Wallin et al., 1994). This initial period could be critical in development which is often a highly precisely timed event in mammals. Targets that are initiated early by *Pax1* could be critical for subsequent gene regulation, such as through the priming of the chromatin landscape or generating essential co-factor proteins required for later regulatory steps. For example, chromatin remodeling and acetylation factors like *Ep300*, *Phf20* and *Hdac2* all gradually declined or increased with the loss of *Pax1* and *Pax9*. As a result, although *Pax9* expression is not dependent on *Pax1*, it may not be able to fully compensate owing to the incomplete chromatin priming and/or lack of initiation of the required co-factor proteins. Certainly these postulations warrant further studies which would illuminate a more detailed mechanism of compensation by these TFs. Performing ChIP-Seq on reciprocal null backgrounds, and identification of Pax1 binding sites in the wild type would help to clarify this hypothesis. Lack of a good ChIP-grade antibody against Pax1 precluded our analysis of Pax1 binding sites in this study.

Remarkably, analysis of our *Pax1/Pax9* regulated targets and *Sox5/Sox6* targets in the IVD revealed the existence of a negative feedback loop between the *Pax* and *Sox* genes. Because *Pax1* and *Pax9* are known to be down-regulated once the pre-chondrogenic cells mature into chondrocytes in the inner annulus (IAF), and the *Sox* trio are essential for and are up-regulated during chondrogenesis (Balling et al., 1988), this negative feedback circuit might explain the initial co-expression of the *Sox* and *Pax* genes in the IVD mesenchyme at E12.5-E13.5 and the subsequent restriction of *Pax1/Pax9* to the OAF by E14.5-E15.5. While *Pax-Sox* relationship is well known in neurogenesis whereby *Pax6* and *Sox1/Sox2* are known to cooperate, here we have identified the existence of such a regulatory axis involving *Pax* and *Sox* in IVD development (Curto et al., 2015). Importantly, this *Pax-Sox* regulation might have a key role in the segregation of IAF and OAF.

Moreover, *Pax1* and *Pax9* positively regulate *Bmp4* and several of the cartilage genes activated by the *Sox* trio. Indeed, Bmp4 is an upstream regulator of the *Sox* trio (Sohn et al., 2010). This prompted us to explore the involvement of BMP pathway in IVD development. Mining the publication by Sohn et al. (2010) revealed that *Pax1/Pax9* regulate genes controlled by BMP and TGF-B pathways in the IVD. At E12.5, *Pax1/Pax9* positively regulate both IAF and OAF genes, while in combination with BMP and TGF-B pathways they appear to be activating IAF genes. We thus posit that *Pax1/Pax9*, BMP and TGF-B pathways together are mainly involved in promoting cartilaginous inner annulus development and preventing inappropriate VB or fibrous annulus development. However, the restriction of *Pax1/Pax9*, BMP4 and TGF-B components to the OAF at E14.5 onwards indicate that they may be involved in subsequent embryonic OAF differentiation. Besides, other signaling pathways may also play a role in this complex process, as the FGF pathway effector, *Etv1*, was downstream of *Pax1/Pax9* and is restricted to OAF at E14.5.

Based on our findings, we propose that *Pax* genes assist in the activation of early chondrogenic genes including *Sox5* and *Bmp4* during the IVD mesenchyme differentiation. In turn, *Sox5* represses *Pax1* and *Bmp4* by negative feedback, allowing the *Sox* trio to take full control of the chondrogenic program. In support of this, the *Sox5^−/−^Sox6^−/−^* IVD mesenchymal cells, which still express *Pax1*, fail to undergo chondrogenesis and remain mesenchymal at E15.5 (Smits and Lefebvre, 2003). Moreover, a recent publication showed that misexpression of *Pax1* in cultured chondrocytes resulted in downregulation of *Sox9*, *Col2a1* and *Acan*, indicating its role as a negative regulator of chondrocyte maturation (Takimoto et al., 2013). While in our study *Pax1/Pax9* positively regulate *Col2a1* and *Acan* in prechondrogenic mesenchymal cells of the IVD, how it regulates these targets during later stages of *in vivo* chondrogenesis, particularly in the context of IVD anlagen, is unknown. Nevertheless, their observation of antagonistic relationship between *Pax1* and *Sox9* support our hypothesis that tipping the fine balance between *Pax* and *Sox* is essential to drive the prechondrogenic IVD anlagen cells towards a cartilaginous inner annulus followed by restriction of *Pax* and *Bmp4* to the fibrous outer annulus in a timely manner.

Our novel findings in this study, of a complex *Pax-Sox* gene network that is interconnected with BMP and TGF-B pathways, illuminates an essential mechanism of the early IVD morphogenesis. Importantly, identification of *Pax1* and *Pax9* as regulators of critical IVD genes has implications in understanding certain forms of kyphoscoliosis that have been linked to human *PAX1* and *PAX9*, as well as degenerative disc disease.

## MATERIALS AND METHODS

### Ethics statement

All animal procedures were performed according to the Singapore A*STAR Biopolis Biological Resource Center (BRC) Institutional Animal Care and Use Committee (IACUC) guidelines which are set by the National Advisory Committee for Laboratory Animal Research (NACLAR) Singapore. The IACUC protocols employed were reviewed and approved by the aforementioned committee before any animal procedures were undertaken for the study described here (IACUC Protocol No: 110689 and 110648). The mouse strains used in this study were housed, maintained and provided by the A*STAR Biopolis Biological Resource Center. The lines described here will be made available to the research community upon acceptance of the manuscript.

### Gene targeting

Bacterial artificial chromosomes (BAC) clone RP24-88N2 and RP24-211J10 (derived from the C57BL/6J mouse strain) containing the *Pax1* (chromosome 2) and *Pax9* (chromosome 12) gene loci respectively were obtained from the BACPAC Resources Centre (CHORI) (bacpacresources.org). According to the manufacturer's protocol, genetic modifications of the clones were performed using the Gene Bridges Quick and Easy BAC Modification kit (#K001, Gene Bridges, Heidelberg, Germany). Details of targeting constructs and mouse generation, and genotyping, Southern blotting, FACS sorting, microarray, ChIP-Seq, histology and primers are described below.

### Targeting strategies using BAC recombineering technology, ES cell homologous recombination and mouse crosses

For the *Pax1^F2A-EGFP:Pax1+^* construct, the *RAKR-GSG-F2A-EGFP-FRT-PGK-gb2-Neo-FRT* cassette was inserted in frame immediately before the STOP codon at exon 5 of *Pax1*. For the *Pax9^EGFP:Pax9–^* construct, the same cassette was inserted at three amino acids after the start of exon 2 of *Pax9*. Successfully modified mutation-free BAC clones were subcloned as per the manufacturer's protocol into a minimal vector using the Gene Bridges Quick and Easy BAC Subcloning kit (# K003, Gene Bridges). A *PmeI* restriction site was added at the end of the lower grabbing arm to facilitate subclone linearization. Linearized subclone was electroporated into R1 or V6.4 mouse embryonic stem cells (ESC) to generate the *Pax1^F2A-EGFP:Pax1+^* and *Pax9^EGFP:Pax9– ^* ES clones respectively (Eggan et al., 2001). Details of the generation of the *Pax1^EGFP:Pax1–^* construct has been described in (Sivakamasundari et al., 2013). The correctly targeted clones were confirmed via Southern blotting, karyotyped and microinjected into 8-cell stage mouse embryos isolated from C57BL/6J mice (Kraus et al., 2010). Owing to post-natal or pre-natal lethality, heterozygote *Pax9^+/–^* mice were mated to generate *Pax9^–/–^* embryos while double heterozygote mice (*Pax1^+/–^Pax9^+/–^*) were mated to generate the *Pax1^–/–^Pax9^+/–^*, *Pax1^+/–^Pax9^–/–^* and *Pax1^–/–^Pax9^–/–^* embryos.

### Southern blotting and PCR genotyping

Methods of genomic DNA extraction from the ESC clones and mouse tail and Southern blotting were described previously (Sivakamasundari et al., 2012). Genotyping details for *Pax1^EGFP:Pax1–^* was performed as described in Sivakamasundari et al. (2013).

### Fluorescence Activated Cell Sorting (FACS) and RNA extraction

Dissociation of mouse embryos into single cells was performed using enzymatic digestion with 0.05% Trypsin (Gibco), DNAse (50 U/ml; Sigma) and Collagenase I & II (100 U/ml; Gibco), followed by filtration through a 100 μM and then a 40 μM cell strainer before centrifugation at 448 ***g*** (2000 rpm) for 5 mins at 4^°^C. The cells were resuspended in 5% fetal bovine serum and 4 mM EDTA in Leibovitz's L-15 medium for cell sorting using FACSAria flow cytometer (BD Biosciences) and collected into a 1.5 ml microcentrifuge tube containing 20% FBS buffer. Gating was performed using E12.5 WT embryos. Sorted cells were spun at 1400 ***g*** for 10 min at 4°C, resuspended in Trizol (Invitrogen) and incubated for 5 min at room temperature (RT). RNA was extracted using Trizol followed by column purification with the QIAGEN RNeasy Micro kit, including on-column DNase treatment, RNA samples were quantified and checked for their integrity using Agilent RNA Pico 6000 Chip and Agilent 2100 Bioanalyzer software according to the manufacturer's protocol. RNA was stored at –80°C until further use.

### Microarray

Purified RNA samples with a RNA integrity number (RIN) value of at least 7.0 were chosen for subsequent cDNA conversion and linear single-round amplification using the NuGEN Ovation^TM^ RNA Amplification V2 kit as per manufacturer's protocol. Biotin labeling was performed with NuGEN Encore^TM^ BiotinIL Module kit. An Illumina MouseWG-6 Expression BeadChip was used for microarray gene expression profiling, performed according to the manufacturer's protocol, except that hybridization was performed at 48°C according to the recommendations by the NuGEN Ovation^TM^ RNA Amplification V2 kit. A minimum of three biological replicates were used for each genotype for microarray analyses. Each biological replicate includes FACS-enriched cells from two finely dissected VCs so as to ensure sufficient RNA for downstream analyses.

### Gene expression analysis using GeneSpring GX 12.5

Illumina® BeadStudio software was used to extract the raw image data from the scanned beadchips. The gene expression data was exported as sample probe profiles in a GeneSpring GX 12.5 compatible text file format, with background subtraction but no normalization. The text file was then imported into GeneSpring GX 12.5 (Agilent) for further gene expression analysis. The raw intensity data was quantile normalized to mitigate any batch effect. All entities were filtered by flags using default criteria in GeneSpring GX 12.5: ‘present’, detection *P*>0.8; ‘absent’, detection *P*<0.6; and ‘marginal’, values in between ‘present’ and ‘absent’. A gene-level analysis was performed and the entities were filtered by expression (including only those that were between 20-100th percentile in at least one out of the total number of samples). Outliers were removed from further analyses. Pair-wise comparison was made with unpaired Student's *t*-test for *Pax1^F2A-EGFP:Pax1+^* (WT) versus *Pax1^–/–^*. For GFP(+) versus GFP(–) comparisons with WT, *Pax1^+/–^* and *Pax1^–/–^*, one-way ANOVA statistical testing was performed. For multiple pair-wise comparisons of WT, *Pax1^–/–^Pax9^+/–^* and *Pax1^–/–^Pax9^–/–^*, Welch, GFP(–) samples were excluded and then one-way ANOVA (unequal variance) statistical testing was employed. Multiple testing correction was performed on the *P*-values with the Benjamini–Hochberg False Discovery Rate (B–H FDR) and Student Newman–Keuls (SNK) post hoc test. All entities with *P*<0.05 and a fold change of ≥1.5 were defined as significant. Functional annotation clustering was performed on the microarray results using DAVID v6.7 (http://david.abcc.ncifcrf.gov/) (Huang da et al., 2009a,b). For GO analyses, ENSMBLE IDs were used, classification stringency ‘medium’.

### Quantitative PCR

Quantitative PCR was carried out using cDNA obtained by NuGEN OvationTM RNA Amplification V2 kit as mentioned above. Maxima SYBR Green/ROX qPCR Master Mix (2×) was used according to manufacturer's protocol with *Gapdh* as endogenous control. qPCR was performed in triplicates and statistical analyses were performed in the Microsoft Excel using the data analysis tool (Analysis ToolPak).

### Gene dosage analysis

All the differentially expressed genes (FC­>1.5 or <–1.5) in the *Pax1^–/–^Pax9^–/–^* versus WT microarray were used in the gene dosage analysis. Microsoft Excel software was used to filter genes for the different groups according to the following criteria: Group1: genes not showing a FC­>1.5 or <–1.5 for WT versus *Pax1^–/–^*, *Pax1^–/–^* versus *Pax1^–/–^Pax9^+/–^* or *Pax1^–/–^Pax9^+/–^* versus *Pax1^–/–^Pax9^–/–^* but only in WT versus *Pax1^–/–^Pax9^–/–^*; Group2: genes not showing a FC­>1.5 or <–1.5 for WT versus *Pax1^–/–^*, or *Pax1^–/–^Pax9^+/–^* versus *Pax1^–/–^Pax9^–/–^* but only in *Pax1^–/–^* versus *Pax1^–/–^Pax9^+/–^* ; and Group3: genes not showing a FC­>1.5 or <–1.5 for WT versus *Pax1^–/–^*, *Pax1^–/–^* versus *Pax1^–/–^Pax9^+/–^* but only in *Pax1^–/–^Pax9^+/–^* versus *Pax1^–/–^Pax9^–/–^*. The normalized intensity values of the genes for each genotype (WT, *Pax1^–/–^*, *Pax1^–/–^Pax9^+/–^* and *Pax1^–/–^Pax9^–/–^*) were averaged across the respective biological replicates and plotted as intensity versus genotype for the respective groups.

### Chromatin Immunoprecipitation (ChIP-Seq)

The VC tissues were dissected from staged (based on M. H. Kaufman morphological criteria (Kaufman, 1992) E12.5 mouse embryos in cold Leibovitz medium. The dissected tissues were homogenized and cross-linked with 1% cross-linking buffer (100 mM NaCl, 50 mM Hepes-KOH, pH 7.5, 1 mM EDTA, 0.5 mM EGTA, 11% formaldehyde) for 10 min at RT. Cross-linking was stopped with 0.25 M glycine and washed once with cold PBS and homogenized again. Nuclear extracts were obtained as per standard chromatin isolation protocol and chromatin was sheared to a size range of 100-500 bp. 2 mg of sheared chromatin was used for pre-clearing in a pre-washed anti-rabbit IgG antibody-conjugated Dynabeads® Protein G (ab46540; Invitrogen; #100.04D) in 0.5% BSA buffer for 1 h at 4°C. 1% of the pre-cleared sample was reserved as input. Immunoprecipitation (IP) was performed using pre-conjugated and pre-washed beads with anti-Pax9 antibody (Santa Cruz) for 24 h at 4°C. After the overnight IP, the beads were washed with wash buffer (50 mM Hepes, 500 mM LiCl, 1 mM EDTA, 1% NP-40, 0.7% Na-Deoxycholate with 1× protease inhibitors) six times followed by one wash with TE buffer (10 mM Tris, pH 8.0, 1 mM EDTA, 50 mM NaCl). The IP chromatin was eluted by incubation at 65°C for 30 min in 210 μl of elution buffer (50 mM Tris-HCl, pH 8.0, 10 mM EDTA, 1% SDS). The eluate and 1% of input that was reserved earlier were de-crosslinked overnight at 65°C DNA, with sequential treatment of RNase A (0.2 mg/ml) and proteinase K (0.2 mg/ml) followed by DNA extraction by standard phenol: chloroform extraction method. DNA was quantified using Picogreen and 15 ng of purified IP or input DNA was used for library preparation using the NEBNext® ChIP-Seq Sample Prep Reagent kit. Size selection of 200-300 bp was performed on a 2% agarose gel and gel purified using QIAGEN gel purification kit. The ChIP DNA libraries were checked for their quantity and quality using Agilent Bioanalyzer DNA 1000 chip kit (#5067-1504) before sequencing on Illumina's Solexa Sequencer. Peak calling using MACS (Zhang et al., 2008), gene ontology and motif analysis methods using Weeder (Pavesi et al., 2004), MEME Suite (Bailey et al., 2009), Centrimo (Bailey and Machanick, 2012) and FIMO (Grant et al., 2011) are described below. Motif databases JASPAR CORE database (Mathelier et al., 2016), the UniPROBE database (Newburger and Bulyk, 2009), Human and Mouse HT-SELEX motifs from Jolma et al. (2013) and HOCOMOCO Human and mouse (v10) were used for motif analyses. Tracks for ChIP-seq peaks were viewed using Integrative Genomics Viewer (Robinson et al., 2011; Thorvaldsdottir et al., 2013) or UCSC genome browser (Kent, 2002).

### Peak calling and motif analysis

After high-throughput sequencing of the short tags of DNA in the libraries, the sequence reads were mapped to the mouse genome (NCBI build 37/mm9). 11,133 peaks were called using Model-based Analysis of ChIP-Seq (MACS) algorithm (Zhang et al., 2008) with the default paremeters and a *P*-value cut-off of 1.00E–10. The following criteria was use to assign the association of peaks to nearby genes: TSS, <1 kb upstream and downstream of the TSS from either side; promoter, 1-5 kb upstream of the TSS; intragenic, >1 kb downstream of TSS within the regions of the gene itself; proximal, 5-10 kb upstream of the TSS and 1-10 kb downstream of the TSS outside the gene; distal, 10-100 kb upstream and downstream of the TSS outside of the gene; others, >100 kb from TSS. The peaks called were ranked based on the *P*-value. *De novo* motif discovery was performed using Weeder (Pavesi et al., 2004) for the top 500 peaks with the lowest *P*-values. Only the central repeat-masked 200 bp sequences in each peak were used to achieve the best performance. Occurrences of the *de novo* discovered motif, Pax9 DBD, Pax1 DBD, Smad1 and Smad2 motifs were searched in all 11,133 peaks using FIMO (Grant et al., 2011) with a *P*-value threshold of 0.001. To identify the enrichment of binding sites of Pax9 and other co-factors, we used CentriMo (Bailey and Machanick, 2012) to scan each Pax9 ChIP-seq peak region (central 500 bp). *De novo* discovered Pax9 motif and all motifs in the JASPAR CORE database (Mathelier et al., 2016), the UniPROBE database (Newburger and Bulyk, 2009), Human and Mouse HT-SELEX motifs from Jolma et al. (2013) and HOCOMOCO Human (v10) and HOCOMOCO Mouse (v10), were used as input motifs. Gene Ontology (GO) enrichment analysis was performed using the Genomic Regions Enrichment of Annotations Tool (GREAT) web-based tool (http://bejerano.stanford.edu/great/public/html/). The default criteria was assigned to define the regulatory domain for this analysis: 5.0 kb upstream, 1.0 kb downstream of the TSS and a 1000 kb (1Mb) extension in both directions to the next closest gene's TSS but a maximum extension in only one direction.

### Genotyping PCR primers

PCR genotyping was performed using the following primers: *Pax1^F2A-EGFP:Pax1+^* Primer pair 1 (F – 5′ CTGTTGAGGAGATCCACTAGCC 3′ and R – 5′ ATCTAAAACCAAGACTCGGAAAGAC 3′; 444 bp) Primer pair 2 (F – 5′ CTGTTGAGGAGATCCACTAGCC 3′ and R – 5′ AGATGAACTTCAGGGTCAGCTTG 3′; 487 bp); and *Pax9^EGFP:Pax9–^* Primer pair 3 (F – 5′ GGGTCTCTCTTCTTGTTTGTTGTT 3′ and R – 5′ CTTGTAAGTCCGGATGTGTTTCAC 3′; 498 bp) Primer pair 4 (F – 5′ GGGTCTCTCTTCTTGTTTGTTGTT 3′ and R – 5′ AGATGAACTTCAGGGTCAGCTTG 3′; 481 bp).

### Primers for qPCR

The primers for qPCR were as follows:

*Gapdh-*F: AACTTTGGCATTGTGGAAGG, R: GGGCCATCCACAGTCTTCT;

*Col2a1*-F: GGCAACAGCAGGTTCACATA, R: CTTGCCCCACTTACCAGTGT;

*Pax1*-F: CCTTGGAGGCAGACATTAAATATAC, R: GTATACTCCGTGCTGGTTGGA;

*Pax9*-F: GCAGTGAATGGATTGGAGAAG, R: GATGCTGAGACGAAACTGCTC;

*Sox9*-F: ACAGACTCACATCTCTCCTAATGCT, R: CTGAGATTGCCCAGAGTGCT;

*Sox5*-F: AGAAACTGCGTATCGGGGAGTA, R: GATGGGGATCTGTGCTTGTT;

*Wwp2*-F: ATCTATCGGCACTACACCAAGAG, R: CCGTGACAAACTGCAGTAGC;

*Acan*-F: CTGCCCTTCACGTGTAAAAAG, R: ACCAGGGAGCTGATCTCGTAG.

### Histology sectioned *in situ* hybridization (SISH) and immunohistochemistry and imaging

Mouse embryos processing methods for histology, SISH and immunohistochemistry were performed as described (Chen et al., 1996; Kraus and Lufkin, 1999; Wang et al., 2000; Zhao et al., 2003). Antibodies and dilutions are as follows: Pax1: SC-25407X, 1:200; Pax9: SC-25410X, 1:200; GFP: SC-9996, 1:50; bovine anti-rabbit IgG-B: SC-2363, 1:400 and horse anti-mouse IgG at recommended dilution by the Vectastain® ABC kit (Vector Laboratories; cat # PK-4002). A LEICA M205 FA microscope was used for fluorescence imaging of embryos and sections were imaged using a Zeiss Axio Imager Z1.
